# Right Dose, Right Now: Development of AutoKinetics for Real Time Model Informed Precision Antibiotic Dosing Decision Support at the Bedside of Critically Ill Patients

**DOI:** 10.3389/fphar.2020.00646

**Published:** 2020-05-15

**Authors:** Luca F. Roggeveen, Tingjie Guo, Ronald H. Driessen, Lucas M. Fleuren, Patrick Thoral, Peter H. J. van der Voort, Armand R. J. Girbes, Rob J. Bosman, Paul Elbers

**Affiliations:** ^1^ Department of Intensive Care Medicine, Amsterdam Medical Data Science (AMDS), Research VUmc Intensive Care (REVIVE), Amsterdam Cardiovascular Science (ACS), Amsterdam Infection and Immunity Institute (AI&II), Amsterdam UMC, Location VUmc, Vrije Universiteit Amsterdam, Amsterdam, Netherlands; ^2^ Intensive Care Unit, OLVG Oost, Amsterdam, Netherlands

**Keywords:** clinical decision support, precision medicine, antibiotic dosing, sepsis, TDM (therapeutic drug monitoring)

## Abstract

**Introduction:**

Antibiotic dosing in critically ill patients is challenging because their pharmacokinetics (PK) are altered and may change rapidly with disease progression. Standard dosing frequently leads to inadequate PK exposure. Therapeutic drug monitoring (TDM) offers a potential solution but requires sampling and PK knowledge, which delays decision support. It is our philosophy that antibiotic dosing support should be directly available at the bedside through deep integration into the electronic health record (EHR) system. Therefore we developed AutoKinetics, a clinical decision support system (CDSS) for real time, model informed precision antibiotic dosing.

**Objective:**

To provide a detailed description of the design, development, validation, testing, and implementation of AutoKinetics.

**Methods:**

We created a development framework and used workflow analysis to facilitate integration into popular EHR systems. We used a development cycle to iteratively adjust and expand AutoKinetics functionalities. Furthermore, we performed a literature review to select and integrate pharmacokinetic models for five frequently prescribed antibiotics for sepsis. Finally, we tackled regulatory challenges, in particular those related to the Medical Device Regulation under the European regulatory framework.

**Results:**

We developed a SQL-based relational database as the backend of AutoKinetics. We developed a data loader to retrieve data in real time. We designed a clinical dosing algorithm to find a dose regimen to maintain antibiotic pharmacokinetic exposure within clinically relevant safety constraints. If needed, a loading dose is calculated to minimize the time until steady state is achieved. Finally, adaptive dosing using Bayesian estimation is applied if plasma levels are available. We implemented support for five extensively used antibiotics following model development, calibration, and validation. We integrated AutoKinetics into two popular EHRs (Metavision, Epic) and developed a user interface that provides textual and visual feedback to the physician.

**Conclusion:**

We successfully developed a CDSS for real time model informed precision antibiotic dosing at the bedside of the critically ill. This holds great promise for improving sepsis outcome. Therefore, we recently started the Right Dose Right Now multi-center randomized control trial to validate this concept in 420 patients with severe sepsis and septic shock.

## Introduction

Antibiotics are essential for the treatment of sepsis and septic shock (de Sousa et al., 2008). The surviving sepsis campaign guidelines (Dellinger et al., 2008) recommend initiation of antibiotic therapy within 1 h of sepsis onset. This recommendation relies strongly on the landmark study by Kumar et al., who showed that every hour of delay of antibiotic treatment following onset of sepsis induced hypotension is associated with a 7.6% mortality increase (Kumar et al., 2006). While causality remain subject of debate, further studies on early goal-directed therapy confirmed the importance of early and appropriate antimicrobial therapy, suggesting specifically that delays after shock recognition should be avoided (Gaieski et al., 2010; Puskarich et al., 2011; Ferrer et al., 2014; Vilella and Seifert, 2014; Sterling et al., 2015; Castaño et al., 2019).

In addition, it is important to consider adequate and timely antibiotic PK target attainment, given the robust and biologically plausible relationship between antibiotic PK exposure and both antimicrobial effect and clinical outcomes (Roberts et al., 2014a; Cardile et al., 2015). This is particularly relevant in the setting of intensive care medicine, where severely ill patients requiring continuous monitoring and support of vital function are treated. For these patients, PK target attainment is challenging throughout their treatment because their pharmacokinetics vary widely and may change vary rapidly due to disease progression as well as therapy (Elbers et al., 2015). The severity of this problem was confirmed by the Defining Antibiotic Levels in Intensive care (DALI) study (Blot et al., 2014; De Waele et al., 2014; Roberts et al., 2014b), observing up to a 500-fold variation in antibiotic concentrations in critically ill patients. Furthermore, less than half of patients achieved the optimal PK target (Roberts et al., 2014b).

With reported mortality rates exceeding 40% for patients with septic shock (Singer et al., 2016; de Grooth et al., 2018; Fernando et al., 2018), it is alarming that most Intensive Care Units (ICUs) continue to rely on standard dosing regimens. This may be related to clinically relevant pharmacokinetic knowledge on antibiotic dosing among intensive care professionals being insufficient (Fleuren et al., 2019). This may explain why, for example, antibiotic PK exposure is rarely increased in the presence of well-known risk factors for underdosing such as young age, large body weight, renal hyperfiltration, and septic shock; and inversely, why antibiotic PK exposure is often not reduced if organ failure develops, potentially giving rise to toxic levels.

Solutions addressing this important clinical challenge include pharmacometric dosing guidance for antibiotics in the form of therapeutic drug monitoring (TDM). However, this approach is often limited to the aminoglycosides and vancomycin, although some centers have investigated TDM for other antibiotic classes including the beta lactams (Muller et al., 2018; Wong et al., 2018). More importantly, the current practice of TDM has major drawbacks. Firstly, TDM requires plasma samples which delay dosing guidance until at least multiple doses have already been administered. Secondly, TDM is not available directly and immediately at the bedside as it relies on data entry, interpretations, and communication by clinical pharmacologists. This may explain why the few clinical studies on TDM that have evaluated relevant clinical outcomes have produced mixed results (Touw et al., 2005; von Gunten et al., 2007; Wong et al., 2014; de Velde et al., 2018).

The last decade has witnessed repeated calls for more personalized antibiotic dosing regimens (Samtani et al., 2010; Roberts et al., 2014a; Cotta et al., 2015; Tängdén et al., 2017; Roberts et al., 2019). These calls are well aligned with our philosophy that personalized antibiotic dosing support should be directly available at the bedside of every critically ill patient. This requires deep integration with the Electronic Healthcare Record (EHR) system and an intuitive simple design with a strong focus on usability. This should enable physicians without advanced knowledge of pharmacometrics to directly adapt antibiotic dosing as necessary for individual patients at all times. This would facilitate rapid and continuous PK target attainment in this vulnerable population and consequently potentially improve patient outcome.

Therefore, we set out to design and implement AutoKinetics, a Clinical Decision Support System (CDSS) for real-time, model informed antibiotic dosing at the bedside of critically ill patients. In this paper we provide a detailed description of its design, development, validation, testing, implementation, and evaluation with a specific focus on how to overcome the many pharmacometric, technical, and regulatory challenges that come with bringing model informed precision dosing to routine clinical care.

## Methods

### Development Team

Based on our prior experience (Elbers et al., 2015) and following multiple discussions with local and external experts in the field of pharmacometrics, information technology, and intensive care medicine, we identified the following roles required to develop AutoKinetics: two intensivists (RB, PE), one clinical information specialist (RD), one pharmacometrician (TG), and three software developers (LR, RD, RB). Together they formed the Right Dose Right Now (RDRN) team. This team received further support from the department of information and communication technology (ICT), the department of Electronic Health Record Support (EVA), and the department of pharmacy at Amsterdam UMC.

### Goal Directed Task Analysis Framework

We used elements of Goal Directed Task Analysis (GDTA) to create a development framework for AutoKinetics. This analysis is based on Endsley's three hierarchical levels of situational awareness and aims to identify which information is needed at what level of integration to make better decisions. As shown in **Table 1**, GDTA led us to subdivide AutoKinetics into separate software modules to facilitate collaborative software development and facilitate EHR integration.

**Table 1:** Division of AutoKinetics by Endsley's levels of situational awareness.

Endsley's Level	Situational awareness	Goal directed task	Expertise
E1	Perception	Real time acquisition and storage of relevant patient data from the EHR.	The IT department, clinical information specialist, ICU physician
E2	Comprehension	A module that incorporation population pharmacokinetic (PK) models from the literature for the construction of a personalized antibiotic dosing advice.	Pharmacometrician, software developer, ICU physician
E3	Projection	Integration of the E2 front-end with the EHR system	The IT department, clinical information specialist, ICU physician

**Figure 1:**
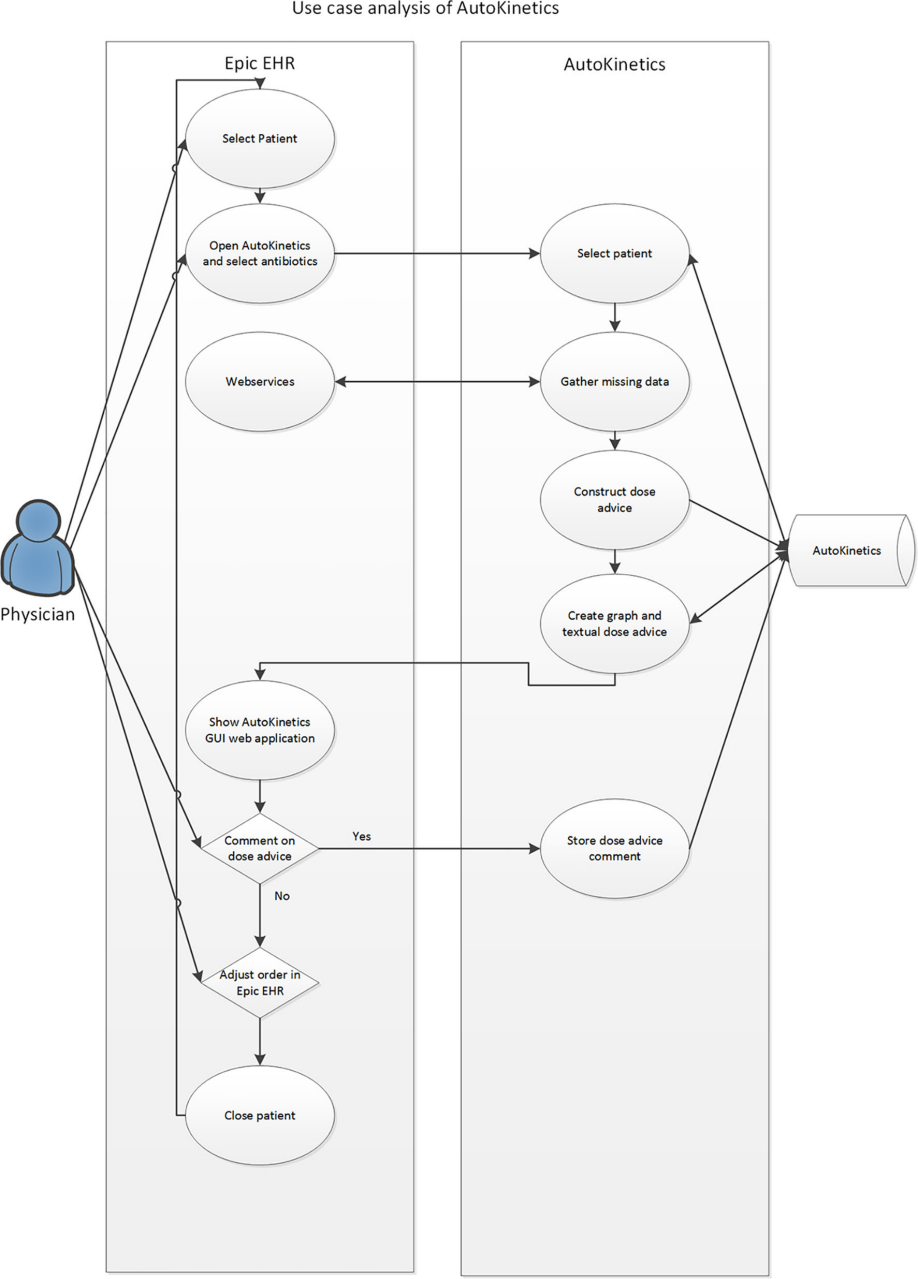
Use case analysis of the interaction of the physician with AutoKinetics through the EHR system.

Repeated round table discussions were held to optimize EHR integration. From the early stages of development, we chose to pursue a passive integration only, meaning that AutoKinetics retrieves data from the EHR and provides a textual and visual advice to the physician but AutoKinetics does not actively change medication orders or sends data back to the EHR. This design serves as a safety “air gap” between the software and the patient. Additionally, it significantly simplifies the integration of AutoKinetics into the EHR. **Figure 1** shows the results of a user interaction analysis performed to assess how ICU physicians would access and operate AutoKinetics through the EHR. Of note, it was our explicit intention that physicians would only need to perform four actions: (1) select patient, (2) select antibiotic, (3) register advice compliance, and (4) change antibiotic order in the EHR system if needed.

**Figure 2:**
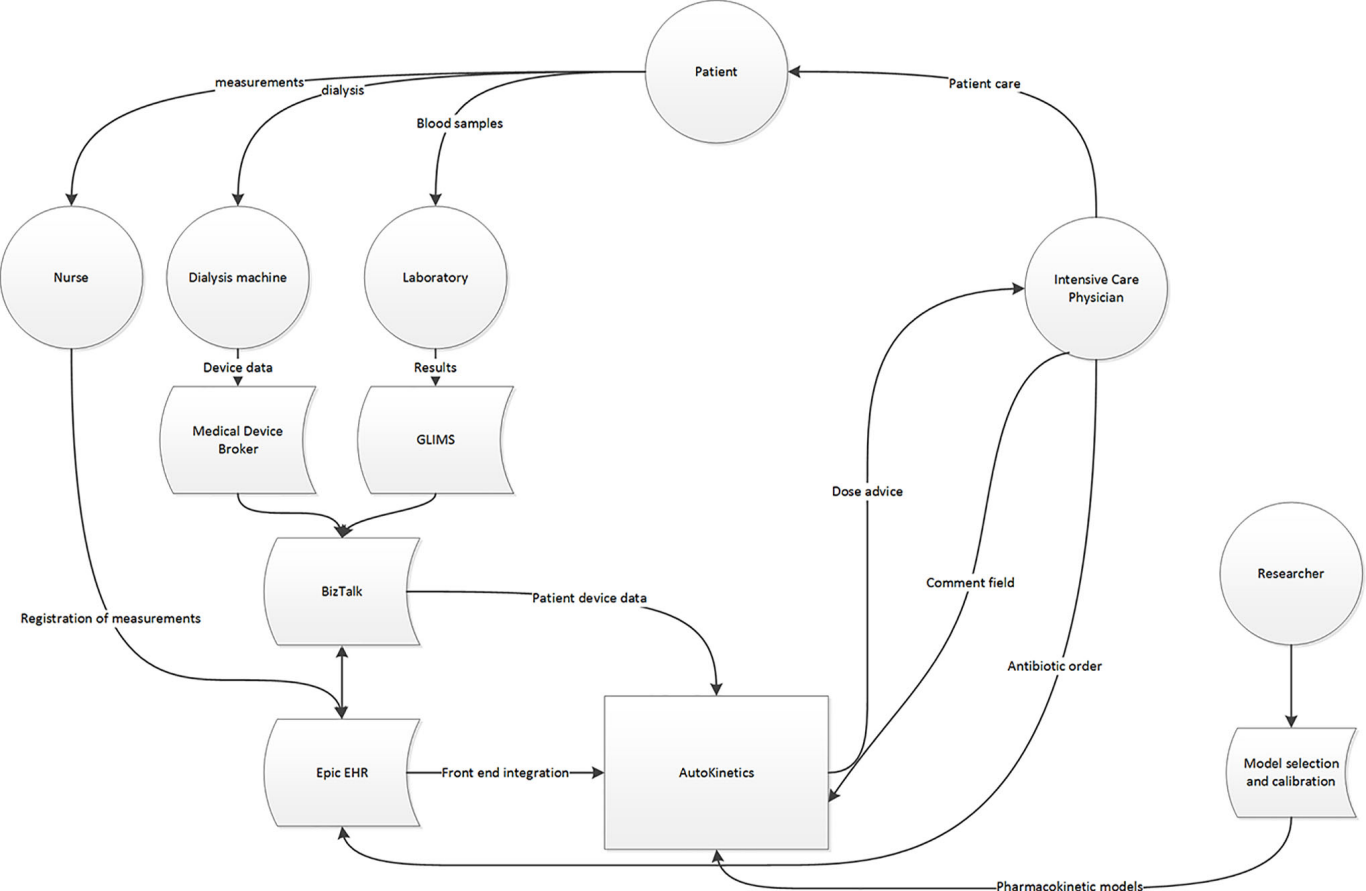
Data stream and user interface analysis to illustrate where patient data originates and travels through the hospital IT infrastructure, the EHR and reaches AutoKinetics for antibiotic dose advice for the physician.

Furthermore, we performed a data stream analysis to better understand the origins and flow through the various IT systems of the required data. **Figure 2** illustrates these data streams and their connections to the EHR and AutoKinetics for identification of points of failure. In this context, the direct connection from the lab system to AutoKinetics is of particular importance as models typically require lab values as input.

### Application Development Life Cycle

**Figure 3:**
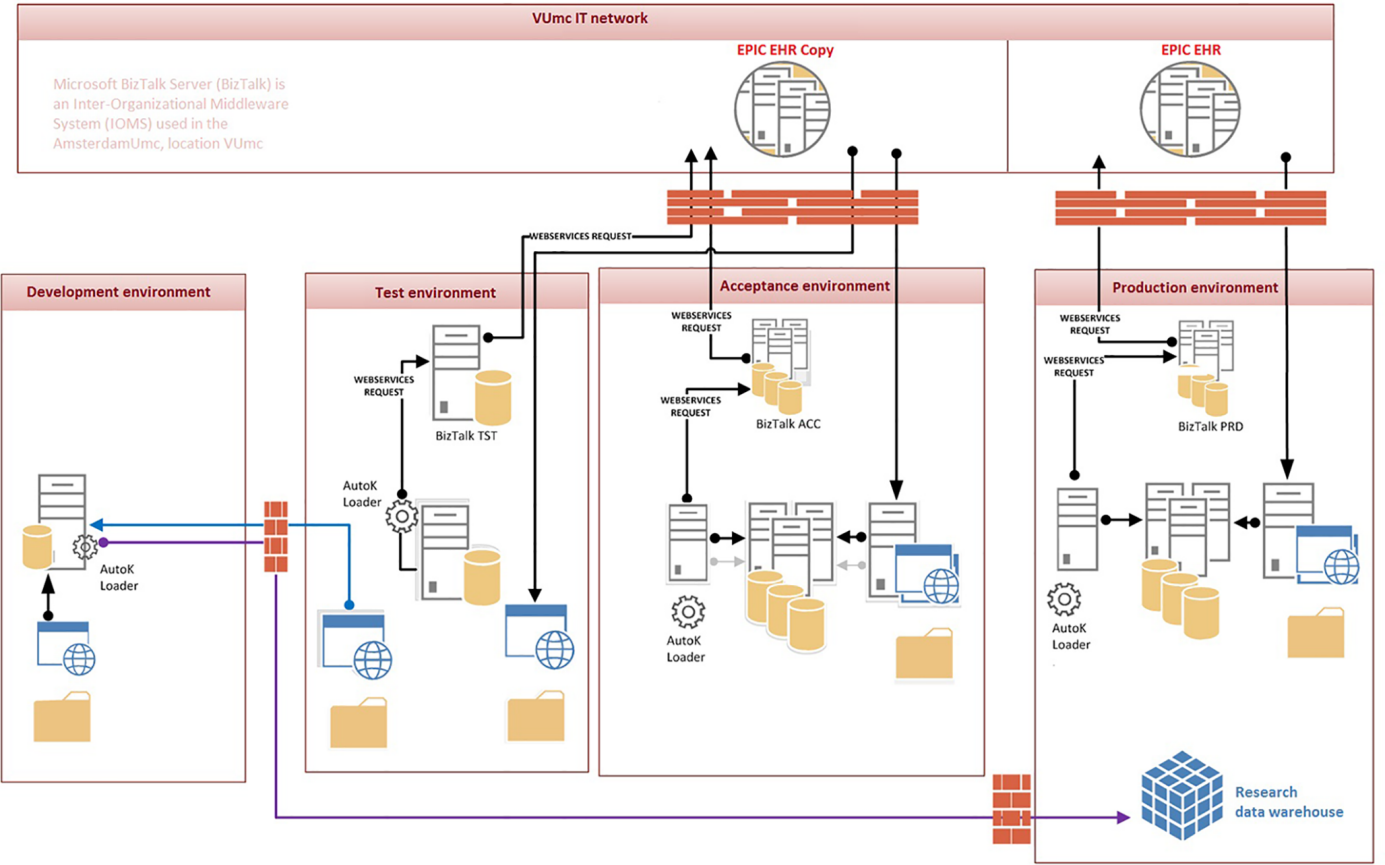
Application development pipeline of AutoKinetics at Amsterdam UMC, location VUmc.

For AutoKinetics to be used in clinical practice, it is vital that regular updates are possible to maintain EHR integration and implement better PK models as these become available. Therefore, we designed an Application Development Pipeline as shown in **Figure 3**. Specifically, we used a four-step process, each with a separate environment, such that real patient data, in a pseudonymized form, was available for developing and testing. This setup allows for thorough refinement of the application to deal with messy, unfiltered, and real patient data ensuring that sufficient data quality checks are in place. The four staging environments each consisted of a typical DTAP street: Development, Testing, Acceptance, and Production. The corresponding development cycle was repeated for each update of AutoKinetics.

### Pharmacokinetic Models

For initial implementation, we chose the five most commonly used antibiotics for the treatment of sepsis in our ICUs, namely meropenem, ciprofloxacin, ceftriaxone, cefotaxime, and vancomycin. Inadequate antibiotic exposure occurs with a frequency of up to 60% for all of these (Blot et al., 2014; Roberts et al., 2014b) and appropriate dosing has been suggested to improve outcomes (Roberts et al., 2014a). We performed a literature search for PK models for the five antibiotics for ICU population. Model performance was validated in retrospectively collected data from our patient population at Amsterdam UMC, location VUmc and from patients at OLVG Oost Hospital, both located in Amsterdam, Netherlands, using a previously published approach (Guo et al., 2019). We also evaluated which models were suitable for clinical use as not all data required by identified models is readily available from the EHR for prospective dosing advice. Selected models were calibrated to further improve the performance prior to being incorporated into AutoKinetics.

### Graphical User Interface Design

To shape the graphical user interface (GUI) we reviewed the literature on CDSS design, focusing on dosing intervention publications within the Healthcare Information Technology (HIT) research field. This wide body of research mostly deals with implementation of EHR based prescribing tools. Frequently, these have been evaluated using surrogate outcomes, such as reducing prescribing errors and safety measures as their primary endpoint (Melton, 2017). A commonly identified underlying cause of failure of HIT implementation is the lack of integration with the clinical workflow (Bowens et al., 2010; Miller et al., 2015; Brown et al., 2017; Melton, 2017).

For example, many EHRs are filled with pop-ups and error messages that interrupt the cognitive process of the physician. This can lead to cognitive and consequently medical errors that pose a danger to patient safety (Schaeffer and Moore, 2012). A systematic review by Brown et al. distilled key themes associated with EHR based prescribing errors that relate closely to human factors and user-centered design and concluded that these errors can be significantly reduced by changing the interface design (Brown et al., 2017). It is clear that a cornerstone in successful implementation of clinical decision support systems is good clinical workflow integration and thoughtful user-centered interface design. Therefore, to develop the GUI for AutoKinetics we conducted several user feedback discussions to assess what functionality and visualizations best suited the clinical decision making process.

### Safety Analysis

The difficulty in analyzing the quality of the AutoKinetics clinical dose advice algorithm is that the advice is dependent on the quality of the PK model used. To mitigate this confounder we empirically validated the AutoKinetics clinical dosing advice strategy by comparing AutoKinetics to current clinical practice including TDM. We compared the actual concentration with the predicted concentration in the first 24 h after a measured plasma level as if the advice by AutoKinetics was followed for that same period using the same available covariate data. All available plasma concentration data including those after the time of advice were used for Bayesian estimation to approximate the so-called true PK profile and forecasted PK profile as if the AutoKinetics advice were followed.

This setup allowed us to compare a pharmacist TDM advice based dosing regimen with the AutoKinetics dosing regimen nearly independent of the quality of the PK model. We used a 24-h time window for evaluation as the AutoKinetics advice would update at least daily using routinely collected data. This approach is inherently limited to patients for whom plasma concentration data are available and TDM was applied. We could therefore use only retrospectively collected data including trough samples from 97 patients that received vancomycin in Amsterdam UMC, location VUmc and OLVG Oost Hospital. For each period and each dosing regimen (pharmacist TDM *versus* AutoKinetics) we calculated the percentage of time that the estimated concentration was within the clinically acceptable range of 10 to 30 mg/L and the AUC_0-24_ was within the clinical desirable range of 400–600 mg*h/L.

Additionally, we performed a risk and safety analysis of the dosing advice generated by AutoKinetics and created safety features to alert the physician to significant deviations from current clinical practice. These features were developed and refined through feedback and discussions with experts in clinical informatics and intensive care medicine.

### Compliance With the Medical Device Regulation and General Data Protection Regulation

The European Union (EU) Medical Device Regulation (MDR, 2017/745) (Ministerie van Volksgezondheid W en S) recently replaced the EU Medical Device Directive. Both specifically require CE certification for medical devices such as AutoKinetics if they are brought to the market AutoKinetics is not yet CE-Certified. Both also allow non CE marked medical devices to be used in the context of clinical research provided national ethical approval is obtained on the basis of an investigational medical device dossier (IMDD), which essentially requires documentation that is quite similar to that required for CE certification. To ensure compliance with the EU General Data Protection Regulation (GDPR, 2016/679) and its national implementation, we engaged the institutional privacy officer. We formally registered the database and we performed a data protection impact analysis as well as an analysis of information and system availability, integrity, and confidentiality, following the requirements outlined in the GDPR.

## Results

### AutoKinetics

AutoKinetics CDSS is a software product written in Microsoft Visual Basic (VB) for the Microsoft Windows operating system (Balena and Fawcette, 1999). A pilot version (Elbers et al., 2015) used the same language while the final prototype was developed in Python 3.6 (Van Rossum and Drake, 1995). We chose VB for the final version to facilitate implementation given the Microsoft oriented hospital IT infrastructure. AutoKinetics consists of five interacting modules that are described in detail below.

#### The AutoKinetics Loader

**Figure 4:**
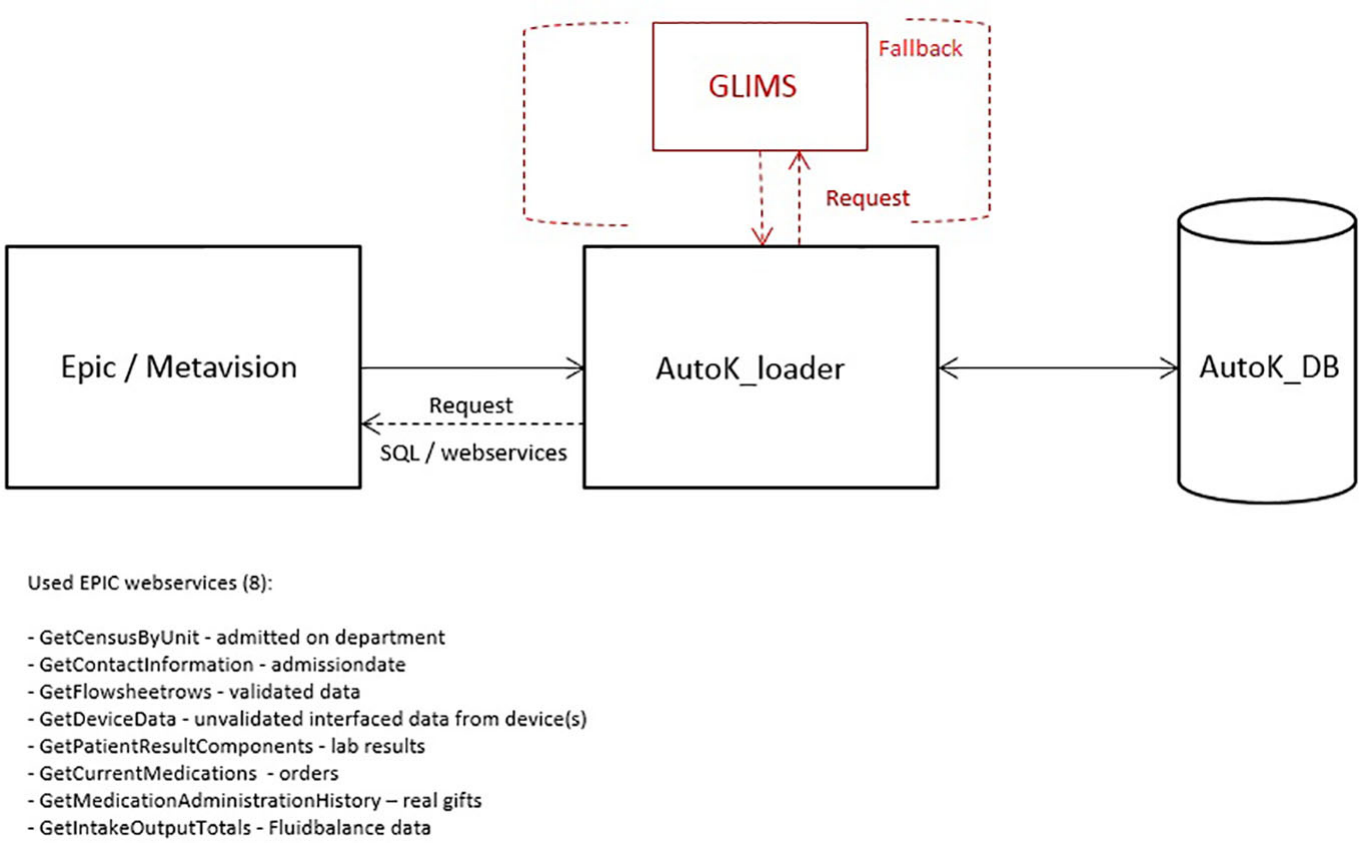
Overview of the AutoKinetics loader (AutoK_loader), data sources, and used EPIC web services used to retrieve patient data as well as the fallback connection to the laboratory database GLIMS.

The AutoKinetics Loader is a microservice which on regular intervals performs a query on the EHR through available web services to extract relevant patient data. We also include a fallback mechanism for laboratory results directly from the laboratory system if the connection to the EHR fails, see **Figure 4**. The data is stored in the AutoKinetics SQL Database. Under normal circumstances, the AutoKinetics Loader runs every 2–5 min with a maximum delay of 5 to 15 min. If no response is provided within 30 min an email warning message is sent to the IT department and the clinical information specialist on call.

#### The AutoKinetics Database

We opted to use a clustered Microsoft Service SQL-database. We chose a clustered setup to ensure data redundancy in case of a database failure and support load balancing of the workload to ensure low latency even during peak usage. The data protection impact analysis identified the use of coded rather than anonymous data as the most prominent risk factor. This was mitigated by ensuring data is kept on premise under national NEN7510 certification.

#### The AutoKinetics Core

This is the main CDSS component which extracts data from the AutoKinetics database, implements the PK model, calculates the required dose, and provides the dosing advice. The three major functionalities of AutoKinetics Core are discussed in detail.

##### Ordinary Differential Equation (ODE) Solver

**Figure 5:**
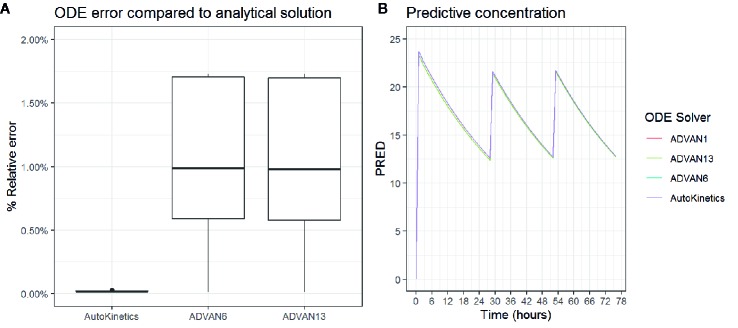
Boxplot of the percentage relative error is estimated concentration **(A)** and Concentration curve for three antibiotic gifts for different ODE solvers **(B)**.

In the case when there is no closed-form solution for PK models, AutoKinetics utilizes a numerical solution for the ordinary differential equation (ODE) system. For the implementation of PK models in AutoKinetics, the well-known Runge-Kutta fourth-order method based ODE solver was implemented (Runge, 1895; Kutta, 1901; Wambecq, 1978). We chose a rather small step size, i.e. time interval of 1 min for the ODE solver in order to be capable of handling any potential stiff systems. To assess the quality of our ODE solver, we empirically compared it to the established ODE solvers implemented in NONMEM^®^ (version 7.4.1; GloboMax LLC, Hanover, MD), which is the gold standard program for PK modelling (Dubovitskaya et al., 2017; Bauer, 2019). The results are shown in **Table 2** and **Figure 5**. The differences in percentage of relative error in the estimated concentration are very small and did not result in different concentration-time curves or dosing advice between NONMEM^®^ and AutoKinetics.

**Table 2:** Comparison of NONMEM^®^ ODE solvers to AutoKinetics.

NONMEM^®^ ODE solver	Median difference in calculated concentration (mg/L) between NONMEM^®^ and AutoKinetics
Analytical solution ADVAN 1 *(suited for one compartment models)*	0.0003344 (0.0001289–0.0007203)
Approximate solution ADVAN 6 (most commonly used ODE solver)	0.0101345 (0.0059696–0.0174771)
Approximate solution ADVAN13 (LSODA method)	0.0100327 (0.0058412–0.0174159)

##### Dose Calculation

The antimicrobial effect of antibiotics depends on their PK exposures, e.g., area under the plasma drug concentration-time curve (AUC) or minimum concentration (C_min_) (Roberts et al., 2014a). The strategy is to reversely find a dose to maintain a needed PK exposure of the target for a chosen dosing interval. Accordingly, AutoKinetics generates dose recommendation based on well-defined PK principles. An important presumption in dose calculation is the linearity of PK. In linear PK, PK exposure remains identical within the same time period regardless of dosing frequency provided that the total dose stays unchanged. For instance, the AUC for a 24 h period (AUC_0-24_) with a once daily 1,000 mg intravenously infused dose of a drug equals the total AUC for twice daily intravenously infused doses of 500 mg. Another implication is that the PK exposure is proportional to the dose. Thus, if one measures the AUC for a 500 mg dose, one can estimate the AUC for a 750 mg dose in the same patient as being 50% greater (Ratain and Plunkett, 2003). Vancomycin, meropenem, and ciprofloxacin usually exhibit linear PK (Ings et al., 1985; Vance-Bryan et al., 1990; Mouton and van den Anker, 1995; Vandecasteele et al., 2013). However, non-linear clearance has been reported for ceftriaxone due to protein binding (McNamara et al., 1982).

###### Maintenance Dose Calculation

Since exposure is proportional to the dose in linear PK, the target maintenance dose (MD) can be calculated using the following equation:

(1) $$MD_{target}=\frac{exposure_{target}}{exposure_{test}}*MD_{test}$$

where MD_test_ and exposure_test_ are the test MD and its corresponding PK exposure and exposure_target_ is the target PK exposure. Two types of PK exposure to determine the PK targets are used in AutoKinetics. For the beta lactams, the percentage of time of the concentration above the minimum inhibitory concentration (MIC) of the bacteria involved (%T > MIC) is used. The area under the concentration-time curve divided by the MIC of the bacteria involved (AUC/MIC) is used for vancomycin and ciprofloxacin.

When the chosen exposure type is %T > MIC, AutoKinetics first simulates the time course of the drug concentration with a random test MD for the antibiotic under consideration. As an example, let us consider a test MD of 400 mg per day. Hence, the C_min_ of test MD can be obtained *via* simulation. If the C_min_ of test MD is 10 mg/L, the target MD for a C_min_ of 20 mg/L can be derived as 20/10*400, which equals 800 mg.

Similarly, for an antibiotic where the antimicrobial effect is related to the ratio of AUC to MIC, AutoKinetics first computes the AUC of a test MD based on the simulated time course of concentration using the trapezoidal rule. By assuming the clearance (CL) of a patient is consistent over the time period of interest, AutoKinetics calculates the target MD through the following equation:

(2) $$MD_{target}=AUC_{target}*CL$$

which does not require simulations of a test MD and is an analytical solution of MD calculation.

###### Loading Dose Calculation

The design of loading dose (LD) calculation in AutoKinetics is to derive a dose that enables the concentration right after the administration to achieve the target maximum concentration (C_max_) of MD-maintained steady state. AutoKinetics simulates the time course of concentration with the precalculated MD given the designated dosing schedule to obtain the target C_max_. This simulation process continues until steady state is reached, which is approximated as after seven half-lives of the drug. Since there could be residual concentration (C_res_) remaining from previous dosing when LD is to be given, the elimination process of C_res_ is considered in order to calculate LD properly.

To achieve this, AutoKinetics relies on the PK superposition principle:

(3) $$f\left(C_{0}\right)=f\left(C1_{0}\right)+f\left(C2_{0}\right)+...+f\left(Cn_{0}\right)$$

where C_0_ represents the total plasma concentration, Cn_0_ represents the nth decomposed plasma concentration, and function f denotes the PK process. This implies that a PK process can be decomposed as the sum of sub PK processes.

We use this to decompose the PK process between start and finish of LD administration into two separate processes: the net elimination of C_res_ and the net increase of concentration due to LD. Thus, AutoKinetics first simulates the time course of C_res_ elimination until LD administration ends, at which moment the concentration, denoted as Ct_res_, can be obtained. Thereafter, the mathematical target maximum concentration of LD equals C_max_ subtracted by Ct_res_ (e.g. C_max_ − Ct_res_). Hence, AutoKinetics simulates a whole administration process for a test LD, following which the target LD can be derived using Eq.1 based on the assumption of linear PK.

##### Clinical Dosing Advice Algorithm

**Figure 6:**
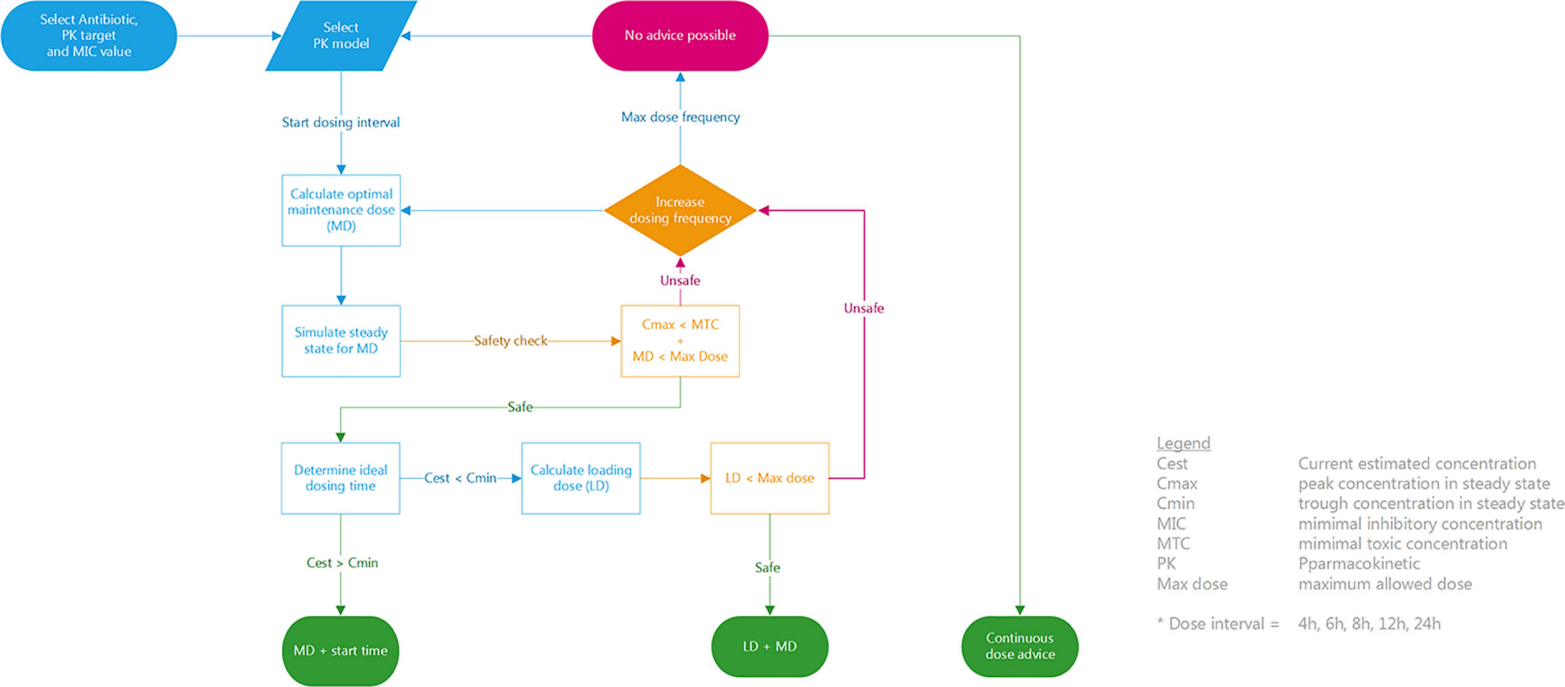
Clinical dosing algorithm to create antibiotic dose advice for AutoKinetics.

The final step in AutoKinetics is to create a dosing advice for a chosen dosing frequency. We have devised a simple, deterministic solution to generate a clinical dose advice using the above dose calculation methods as shown in **Figure 6**. It is up to the Physician to select the antibiotic and AutoKinetics will select the appropriate corresponding PK model. Each antibiotic has a predefined dose interval from baseline (e.g. once daily or three times daily) up to six times daily. AutoKinetics will iteratively cycle through dosing intervals and for each dose interval calculate a corresponding MD and LD. If the calculated maintenance and if needed loading doses are deemed safe, a dose advice will be generated to either achieve steady state immediately (with a LD) or advice solely a MD and an estimated ideal dosing time to maintain steady state. If no safe advice can be generated, a continuous dose advice will be calculated if that option is turned on in the AutoKinetics database. When no advice can be provided the user is recommended to contact the AutoKinetics support team or the pharmacist.

#### The AutoKinetics Bayesian Maximum A Posteriori Calculator

For some antibiotics, specifically for vancomycin, model-based TDM is currently used in clinical practice to adjust individualized dosing regimens. We created a module for AutoKinetics to apply Bayesian estimation, like most TDM software, if plasma concentration data are available. We apply a correction factor to each of the PK model parameters (clearance and volume) that are associated with random-effect parameters in the model. AutoKinetics constructs a posterior density function with respect to the correction factor vector and executes maximum a posteriori (MAP) estimation. During the MAP estimation process, a balance has to be struck between the adjustment of the correction factors and individual goodness of fit of the concentration, which are conflicting with each other. There is a time related computational limitation on the search of the parameter space under consideration if the dose advice is to be used in real time. Therefore, we apply the Simulated Annealing method, which is an adaptation of the Metropolis-Hastings algorithm, to find the estimates of correction factors based on a total of 5,000 times iterative search.

#### The AutoKinetics Graphical User Interface (GUI)

**Figure 7:**
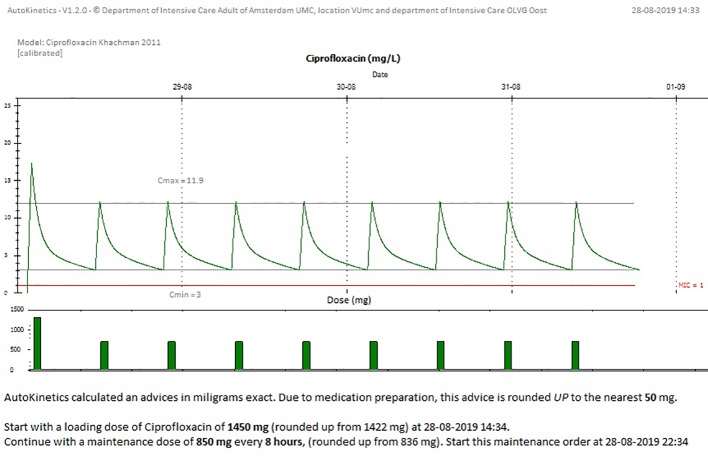
Screenshot from AutoKinetics presenting a dosing advice for Ciprofloxacin, incorporating TDM.

We developed two graphical user interfaces for AutoKinetics, one windows application and one web browser based solution. They share the same visuals and text. We chose a minimalistic style for the visualization of AutoKinetics. We wanted to strike a balance between density of information, which increases mental strain, and the added value of that information in the decision making process. The goal of our design was to provide the physician with all necessary information, but not more, in order for them to be able to accurately interpret the graph even if they would have only minimal pharmacometric knowledge. As can be seen in **Figure 7**, we provide two graphs with a shared X-axis (time), colors are used to differentiate between past (blue) and future (green and red) for the antibiotic concentrations and doses. We provide the physician with a visual representation of the predicted concentration curve assuming the advice, consisting of a dose, dosing interval, and start time, is followed. If available, measured concentrations are included in the plot with a red dot.

### Pharmacokinetic Models

The literature search was performed in December 2017 and a total of 18 models were identified and selected for evaluation for four of the five chosen antibiotics: nine for meropenem, six for vancomycin, three for ciprofloxacin, and one for ceftriaxone. No intensive care PK models were found for cefotaxime. As no suitable model was found for cefotaxime, a new one compartment model was developed on 50 patients from the OLVG Hospital. Covariates effects were analyzed through forward selection and backward elimination procedure. Albumin, Sequential Organ Failure Assessment (SOFA) score, and serum creatinine level were identified as covariates that influenced clearance. No covariate was found to influence volume of distribution. The full model development for cefotaxime and validation of candidate models is available in the **Supplementary Appendix**.

**Figure 8:**
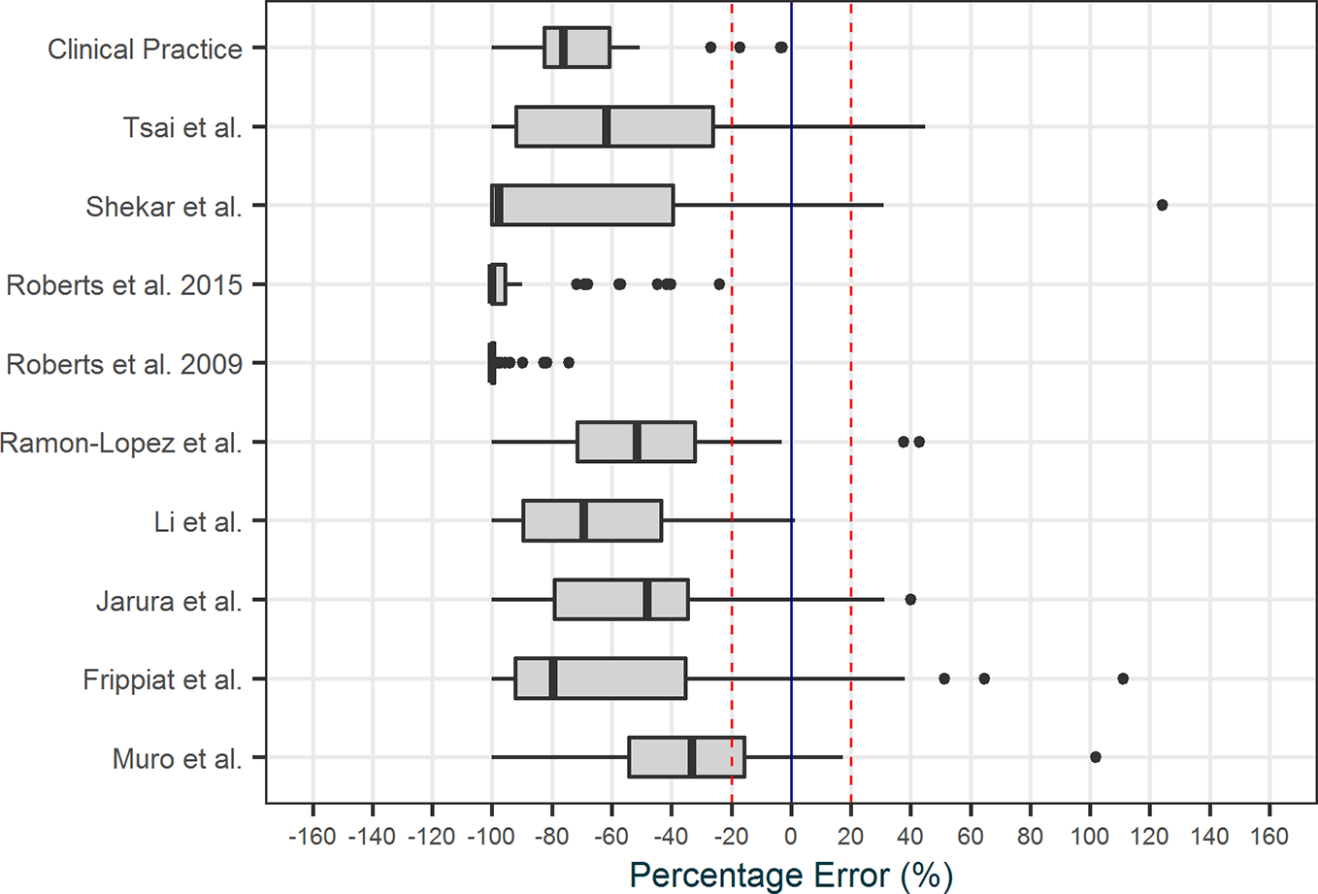
Prediction error plot of the candidate models for Meropenem.

All models that were implemented in AutoKinetics are given in **Table 3**. These models are either one or two compartment models and were developed using NONMEM^®^. Model validation results for vancomycin have been published previously (Guo et al., 2019). Chosen models for implementation were further calibrated by updating the typical values of the PK parameters to better fit our data. A summary of the evaluation of candidate models for the other antibiotics from the literature review is provided separately in the **Supplementary Appendix**. Models from literature were evaluated using Goodness-of-fit plots and by comparing prediction error. As an example, the prediction errors of candidate models for meropenem are shown in **Figure 8**.

**Table 3:** Implemented PK models for ICU Patients.

Antibiotic	Model	Pubmed ID	CMT	CL	V1	Q1	V2	IIV	Residual error	Software	Algorithm	COVARIATES
Meropenem	Muro 2011 n=68	21366653	1	11.1 L/h	33.6 L	NA	NA	Exponential	Add	NONMEM	FO	$CL=\theta_{CL}\times {\left(\frac{mSCR}{0.7}\right)}^{\theta_{mSCE\_CL}}$
												*θ*_*CL*_=11.1 *θ*_*mSCR*_*CL*_ =−1 (if SCR < 0.4 mg/dL, mSCR=0.4)

Ciprofloxacin	Khachman	21653603	2	18 L/h	38 L	60 L/h	73 L	Exponential	Prop	NONMEM	FOCE+I	$CL=\theta 1\times {\left(CL_{Cr\;Cockcroft}/91.7\right)}^{\theta 2}$
	2011											
	n=102											
Ceftriaxone	Garot 2011 n=54	21545483	2	0.56 L/h	10.3 L	5.28 L/h	7.35 L/h	Exponential	Prop	NONMEM	FOCE	$CL=\theta_{CL}+\theta_{CLCr}\times \frac{CLcr}{4.26}\text{Cockcroft}-\text{Gault}(\mathrm{as instead})$
												*θ* *CL* = 0.56 *θ*_*CLCr*_ = 0.32

Vancomycine	Roberts 2011 N=206	21402850	1	4.58 L/h	1.53 L/kg	NA	NA	Exponential	Add, Prop	NONMEM	FOCE+I	$CL=\theta_{CL}\times \frac{CL_{cr}}{100}$
												*θ*_*CL*_ = 4.58
												*V*1 = *θ*_*V*1_ × *BW* *θ*_*V*1_ = 1.53

Add, Additive residual error; CL, Clearance of central compartment; CMT, Number of compartments; FO, First order estimation; FOCE, First order conditional estimation; IIV, Inter individual variability; n, Number of patient used for model development; NA, Not applicable; NCA, Non compartmental analysis; NPAG, Non Parametric Adaptive Grid; Prop, Proportional residual error; Q1, Inter-compartmental clearance between central and peripheral compartments; V1, Volume of distribution of central compartment; V2, Volume of distribution of peripheral compartment.

### Safety Analysis

**Figure 9:**
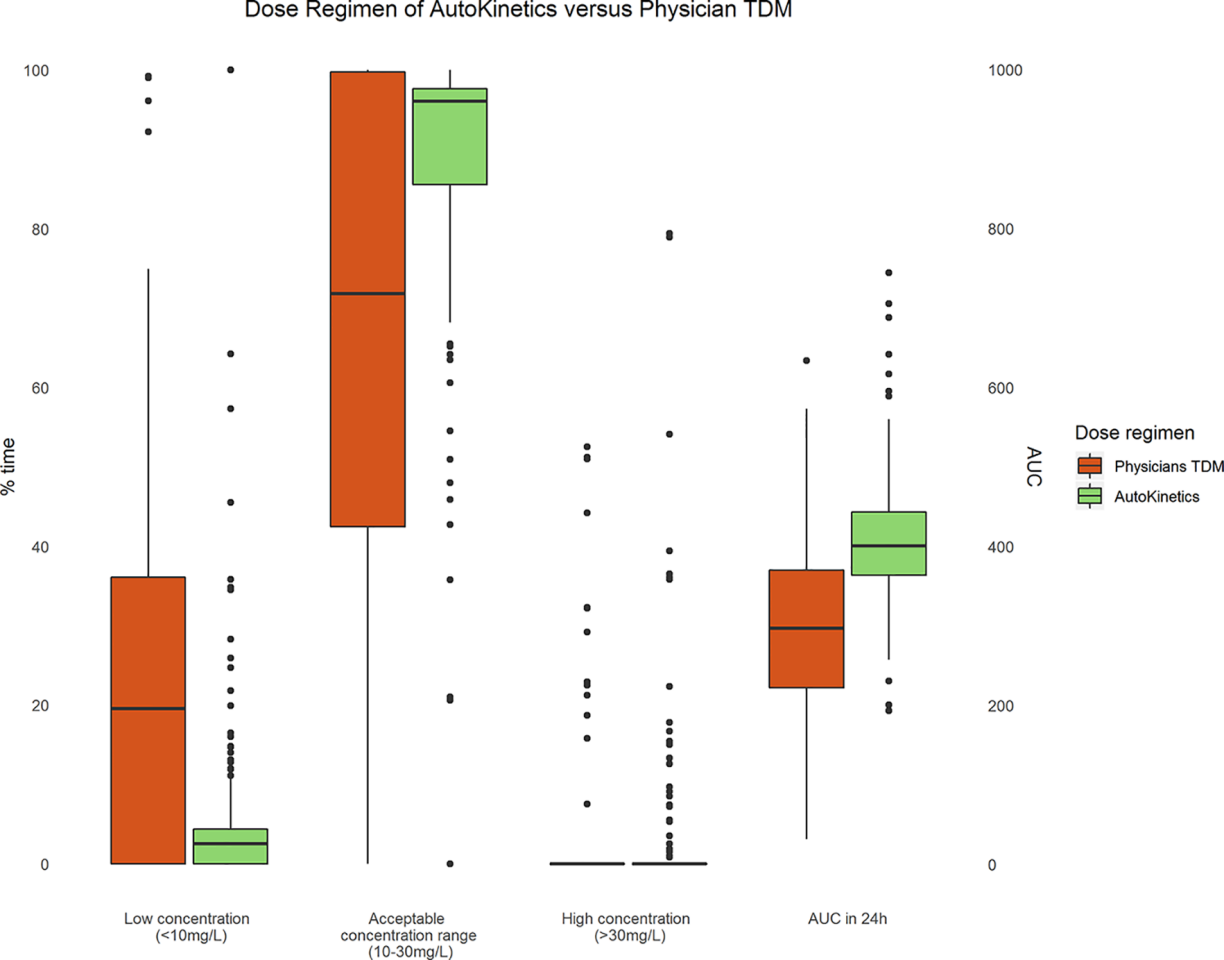
Paired boxplots of the percentage of time (left y-axis) patients are within a concentration range and AUC (right y-axis) of the first 24 h after a measured plasma level for Physician TDM and simulated AutoKinetics dose regimen.

**Figure 9** shows boxplots comparing AutoKinetics versus pharmacist TDM dosing strategies. The AutoKinetics dosing regimen would have led to an increase in percentage of time patients remain in the desired concentration range of 10–30 mg/L. Furthermore, AutoKinetics would have led to a reduction in percentage of time patients are below (< 10 mg/L) the desirable range. Additionally, AutoKinetics would have increased the number of patients who reach the AUC_0-24_ range of 400 to 600 mg*h/L.

Before implementing AutoKinetics in clinical practice we analyzed the risk this would impose on patients and introduced safety measures. In the context of antibiotic dosing support, it is important to differentiate between the risks imposed by: (1) using PK models and (2) using a clinical decision support system to facilitate the use of PK models.

1. Use of Pharmacokinetic Models
  Firstly, PK models should be carefully selected to fit the target patient population and undergo evaluation and calibration before implementation as has been performed for AutoKinetics. In current routine practice, dosing support by PK models in individual patients is not applied in our hospitals with the exception of vancomycin for which conventional TDM is used. While the use of PK models would theoretically lead to improved antibiotic PK target attainment, using PK models could also lead to dosing lower or higher than desired antibiotic plasma levels. If done properly, using a model should always be safer than using no model at all. Nonetheless, the effect of population PK based individualized dosing requires further investigation. A prospective clinical trial, is necessary before widespread implementation. We are currently conducting such a trial.
2. The Use of Software to Facilitate the Use of Pharmacokinetic Models
  For each antibiotic AutoKinetics has built in upper limits, defined as the max dose and minimal toxic concentration, for acceptable MD and C_max_ respectively in the clinical dose advice algorithm. The clinical dosing algorithm underwent a safety analysis, showing a potential increase in PK target attainment. Even so, the risk of model-based dosing can best be mitigated by measuring plasma concentration and consequent dose correction for which AutoKinetics has built in functionality. We have created several secondary lines of defense for AutoKinetics to mitigate the risk of higher and lower than desired plasma concentrations.

First, by design and so by definition, the decision whether or not to follow a dosing advice is always at the discretion of the treating physician, who has to actively place an order in the EHR. For safety reasons, the software is not designed to provide automatic closed loop dosing.

Second, on screen textual warnings are issued whenever a dosing advice is proposed that would lead to more than twice or less than half of the standard dose over 24 h. This advice may be less reasonable, so this should prompt review by the treating physician. The contact number for the AutoKinetics support team and ICU pharmacists are provided to facilitate discussion should the physician so desire. Based on this review, the physician may of course decide not to follow the advice.

Third, if the data used for model is not as recent as is expected from routine clinical practice (e.g. Creatinine is measured at least every 24 h) a textual warning is provided to the physician that older than recommended data is being used which should prompt review of the advice.

Finally, we deliberately chose to provide the physician with an advice that is independent of the current antibiotic order, meaning we do not provide a relative advice (e.g. increase dose by 200 mg). The reasoning for the decision is twofold. First, it leaves to the physician the decision how to adjust the order in the EHR system and does not assume any prior knowledge of the physician of the current antibiotic order. Second, there is an inevitable delay in order entry into the EHR and order extraction by the AutoKinetics loader. Consequently, the relative advice would be incorrect if the updated antibiotic order was not yet processed by AutoKinetics. An independent advice is therefore the safest solution.

## Discussion

To the best of our knowledge, AutoKinetics is the first decision support tool that provides instant bedside antibiotic dosing advice for critically ill patients. Novelties of the AutoKinetics approach include its direct availability at the bedside, its readily accessibility through the EHR and its complete avoidance of any manual data entry. Thus, dosing guidance is immediately available whenever physicians need it.

This implies that the dosing advice provided by AutoKinetics is directly actionable as it does not require any additional tools or support staff. This also means that dosing advice is available even before the first dose is given and that there is no need to wait for plasma samples to adjust dosing. However, Bayesian estimation is available directly through AutoKinetics should plasma levels be available. In addition, AutoKinetics provides real time graphical feedback on proposed dosing, which may enhance pharmacokinetic knowledge among healthcare professionals.

It is remarkable that despite repeated calls for more personalized antibiotic dosing strategies, no randomized controlled trials have been conducted on the matter. Likewise, little has been written about personalized software dosing tools for antibiotics. In 2013, Fuchs et al. (2013) were the first to perform a review of the available software solutions for therapeutic drug monitoring and found 18 different software's. Most were purely academic and research solutions. Furthermore, these tools need considerable technical expertise to use (Al-Metwali and Mulla, 2017). Only two commercial software applications for use in clinical practice beyond their research center were available. (Fuchs et al., 2013) Fuchs's review was revisited in 2018 by Drennen et al. and they noted that “Bayesian TDM software emerged from pharmacometric research units and occupied a transitional space between research and clinical practice” but bedside individualized dose adjustments were still just a “common marketing promise” (Drennan et al., 2018).

The biggest challenge that hinders bedside implementation of personalized dosing is integration with EHR systems. Data retrieval and processing remain burdensome, mostly manual, tasks in current commercial applications. We believe that it is a considerable advantage that some project members took multiple roles. In addition, the physical workplaces of these core team members were positioned in the closest possible proximity to each other to facilitate collaboration. This led to a fast development cycle and good integration within the clinical workflow. In comparison, the developers of TUCUXI, a recently developed TDM software, identified 19 steps to perform TDM in clinical practice until a dose adjustment is made. They were able to reduce this to 10 steps while also reducing manual workload (Dubovitskaya et al., 2017). Nonetheless, the need for a trained operator, antibiotic plasma levels and several manual steps imply the lack of bedside available dose advice limit the potential of this TDM software. Using AutoKinetics, the physician only needs to select the patient in the EHR system, open AutoKinetics, and select the antibiotic to get a dose advice and a final step to adjust to antibiotic order. Thus, a meager four steps are taken by the physician and can be performed immediately when the physician desires. This theoretically leads to a reduction in time until dose adjustment and might therefore significantly improve overall PK target attainment. The results of the simulation performed in this study provide empirical support for this premise by showing a potential increase in time patients are within a desirable therapeutic range.

Considering that the two most difficult aspects of personalized dosing, automatic data retrieval, and automatic advice generation, have been addressed by AutoKinetics, the possibilities to scale AutoKinetics to a wider audience are extensive. Currently, AutoKinetics is available to ICU physicians only. First, within the same hospital and EHR system, AutoKinetics can also be used for non-critically care patients as long as suitable PK models are selected from literature or appropriately developed in target patient population. Second, future integration of AutoKinetics into other hospital EHR systems will allow implementation in other ICUs for a wider range of critically ill patients. Another advantage of automatic data retrieval is that models with many more covariates could be considered. This may improve dosing for individual patients as the model would better describe widely varying PKs. However, this also risks model overfitting, so a balance should be sought. Finally, AutoKinetics could also be used to combat the threat of antimicrobial resistance by reducing the time antibiotic concentrations remain in the mutant selection window (Mouton et al., 2011).

Currently, there are also certain limitations to AutoKinetics. First, it has limited functionality by only providing a dose advice. As such, it does not inform the physician of the correctness of the choice of antibiotics or the appropriate duration of the treatment course. We intend to include such decision support in the future. Second, AutoKinetics is only as good as the quality of its PK models. AutoKinetics support the implementation of population PK models. The field of pharmacometrics is evolving rapidly and more sophisticated models (e.g. physiologically-based PK models) are under development. To also support the state of the art of the near future, AutoKinetics would need to also support more complex models designs. Third, AutoKinetics does not provide closed-loop antibiotic dosing and consequently a human factor is still involved. This has a clear safety advantage but also limits the speed and possibly even quality at which appropriate dose corrections are carried out. If proven safe and effective it is not inconceivable that nurses are trained to use AutoKinetics under the supervision of the physician with the support of the pharmacist for dose advice for those complex patients for whom AutoKinetics cannot provide one. Last, pharmacodynamic information, which could be of major interest for end users, is not readily available from the current interface although such information is intrinsically calculated and MIC values may be directly retrieved from the electronic health record.

## Conclusion

We successfully developed a clinical decision support system for real time model informed precision antibiotic dosing at the bedside of the critically ill. We hypothesize that our solution may contribute to improved antibiotic dosing in the critically ill. If proven feasible and successful, AutoKinetics would provide the necessary clinical support for individualized antibiotic dosing and could be a major step forward in the treatment of sepsis, which is a significant source of worldwide morbidity and mortality. Therefore, we recently started the Right Dose Right Now multi-center randomized control trial to validate this concept in 420 patients with severe sepsis and septic shock.

## Data Availability Statement

The datasets generated for this study are available on request to the corresponding author.

## Ethics Statement

For pharmacokinetic model evaluations for use in AutoKinetics approval was sought and obtained from the Medical Ethics Committee at Amsterdam UMC, location VUmc (2017.018; 2017.282 and 2017.152). The patients/participants provided their written informed consent to participate in this study.

## Author Contributions

PE and RB conceived the presented idea. LR and TG contributed equally to the entire project including design, software development, and implementation of AutoKinetics and wrote the manuscript. RD, RB, and PT contributed to the software development and EHR integration of AutoKinetics. PV and AG facilitated EHR integration and contributed to the privacy framework of AutoKinetics. LF aided in interpreting the results and worked on the manuscript. All authors discussed the results and contributed to the final manuscript.

## Funding

This study is partly funded by the Rational Pharmacotherapy program by ZonMW, The Netherlands Organisation for Health Research and Development (Project number 80-83600-98-40050). Funders had no role in the study design or proceedings, writing of the report, or the decision to submit the report for publication.

## Conflict of Interest

The authors declare that the research was conducted in the absence of any commercial or financial relationships that could be construed as a potential conflict of interest.
