# Supplementary material for: Right Dose, Right Now: Development of AutoKinetics for Real Time Model Informed Precision Antibiotic Dosing Decision Support at the Bedside of Critically Ill Patients
Source: Front Pharmacol. 2020 May 15;11:646. doi: 10.3389/fphar.2020.00646 (PMC7243359; doi:10.3389/fphar.2020.00646)
Supplement: Supplementary file 1 [file DataSheet_1.docx]

**APPENDIX**

**Literature search**

A literature search was performed to identify potential pharmacokinetic models for critically ill patients. A summary of identified models of this literature review is provided separately in the supplementary materials. The following search queries were used in Pubmed to identify PK models for 4 antibiotics: Ceftriaxon, Meropenem, Ciprofloxacin and Cefotaxime. Results of the model validation and model selection for Vancomycine have been reported previously and available at DOI:10.1128/AAC.02543-18.

Meropenem: ("meropenem"[Supplementary Concept] OR "meropenem"[All Fields]) AND (pharmacokinetic[All Fields] OR ("pharmacokinetics"[Subheading] OR "pharmacokinetics"[All Fields] OR "pharmacokinetics"[MeSH Terms]) OR model[All Fields]) AND (("critical care"[MeSH Terms] OR ("critical"[All Fields] AND "care"[All Fields]) OR "critical care"[All Fields] OR ("intensive"[All Fields] AND "care"[All Fields]) OR "intensive care"[All Fields]) OR ("critical care"[MeSH Terms] OR ("critical"[All Fields] AND "care"[All Fields]) OR "critical care"[All Fields]))

Ceftriaxone: ("ceftriaxone"[MeSH Terms] OR "ceftriaxone"[All Fields] OR "ceftriaxone"[All Fields]) AND (pharmacokinetic[All Fields] OR ("pharmacokinetics"[Subheading] OR "pharmacokinetics"[All Fields] OR "pharmacokinetics"[MeSH Terms]) OR model[All Fields]) AND (("critical care"[MeSH Terms] OR ("critical"[All Fields] AND "care"[All Fields]) OR "critical care"[All Fields] OR ("intensive"[All Fields] AND "care"[All Fields]) OR "intensive care"[All Fields]) OR ("critical care"[MeSH Terms] OR ("critical"[All Fields] AND "care"[All Fields]) OR "critical care"[All Fields]))

Ciprofloxacin: ("ciprofloxacin"[MeSH Terms] OR "ciprofloxacin"[All Fields] OR "ciprofloxacin"[All Fields]) AND (pharmacokinetic[All Fields] OR ("pharmacokinetics"[Subheading] OR "pharmacokinetics"[All Fields] OR "pharmacokinetics"[MeSH Terms]) OR model[All Fields]) AND (("critical care"[MeSH Terms] OR ("critical"[All Fields] AND "care"[All Fields]) OR "critical care"[All Fields] OR ("intensive"[All Fields] AND "care"[All Fields]) OR "intensive care"[All Fields]) OR ("critical care"[MeSH Terms] OR ("critical"[All Fields] AND "care"[All Fields]) OR "critical care"[All Fields]))

Cefotaxime: ("cefotaxime"[MeSH Terms] OR "cefotaxime"[All Fields]) AND (pharmacokinetic[All Fields] OR ("pharmacokinetics"[Subheading] OR "pharmacokinetics"[All Fields] OR "pharmacokinetics"[MeSH Terms]) OR model[All Fields]) AND (("critical care"[MeSH Terms] OR ("critical"[All Fields] AND "care"[All Fields]) OR "critical care"[All Fields] OR ("intensive"[All Fields] AND "care"[All Fields]) OR "intensive care"[All Fields]) OR ("critical care"[MeSH Terms] OR ("critical"[All Fields] AND "care"[All Fields]) OR "critical care"[All Fields]))

Supplementary Table 1. Overview of evaluated PK models for ICU Patients

| **Antibiotic** | **Model** | **Pubmed ID** | **CMT** | **CL** | **V1** | **Q1** | **V2** | **IIV** | **Residual error** | **Software** | **Algorithm** | **COVARIATES** |
| --- | --- | --- | --- | --- | --- | --- | --- | --- | --- | --- | --- | --- |
| Meropenem | Tsai  2016  n=11 | 27771187 | 2 | 14.1 L/h | 13.6 L | 20.264 L/h | 8.514 L | Not reported | Not reported | Pmetrics | NPAG | $CL=\theta_{CL}\times\frac{{CL}_{Cr}}{100}$  $\theta_{CL}=14.1$ Cockcroft-Gault (as instead)  $V1=\theta_{V1}\times\left( \frac{BW}{80} \right)^{0.75}$  $\theta_{V1}=13.6$ |
| Meropenem | Roberts  2015  n=24 | 25881576 | 1 | 38 L/h | 17.5 L | NA | NA | Exponential | Add, Prop | NONMEM | FOCE+I | NA |
| Meropenem | Shekar  2014  n=21 | 25636084 | 2 | 5.1 L/h | 18.7 L | 21 L/h | 13.2 L | Exponential | Add, Prop | NONMEM | FOCE+I | $CL=\theta_{CL}\times{CL}_{RRT}+\theta_{CL}\times\left( {CL}_{NORRT}\times CrCL \right)$  ${CL}_{RRT}=0 for patients not receiving RRT$  ${CL}_{NORRT}=0 for patients receiving RRT$  $CrCL=Cockcroft-Gault$ |
| Meropenem | Ramon-Lopez  2015  n=12 | 25362574 | 2 | 0.196 L/h/kg | 0.273  L/kg | 0.199 L/kg/h | 0.309  L/kg | Exponential | Prop | NONMEM | FOCE+I | $CL=\theta_{CL}\times\left( 1-\theta_{AGE_{CL}}\times(AGE-46) \right)\times\left( 1-\theta_{albumin}\times(albumin-15) \right)$  $\theta_{CL}=0.196 \theta_{AGE_{CL}}=0.023 \theta_{albumin}=0.049$  $V1=\theta_{V1}\times\left( 1-\theta_{albumin}\times(albumin-15) \right)$  $\theta_{V1}=0.273$  $V2=\theta_{V2}\times\left( 1-\theta_{albumin}\times(albumin-15) \right)$  $\theta_{V2}=0.309$ |
| Meropenem | Frippiat  2015  n=55 | 25216821 | 2 | 10.2 L/h | 5.2 L | 6.9 L/h | 12.1 L | Exponential | Add, Prop | NONMEM | FOCE+I | $CL=\theta_{CL}\times\left( \frac{GFR}{GFRmean} \right)^{\theta_{GFR\_CL}}$  $\theta_{CL}=10.2 \theta_{GFR\_CL}=0.73$ MDRD  $V1=\theta_{V1}\times\left( \frac{BW}{BWmean} \right)^{\theta_{BW\_V1}}$  $\theta_{V1}=5.2 \theta_{BW\_V1}=0.6$  $Ve=11.3$  $\theta_{ce}=66.5$ |
| Meropenem | Roberts  2009  n=10 | 19398460 | 2 | 13.6 L/h | 7.9 L | 56.3 L/h | 14.8 L | Exponential | Add, Prop | NONMEM | FOCE+I | $CL=\theta_{CL}\times fCG$ renal function described using the Cockroft–Gault equation normalized to 6 L/h (fCG)  $\theta_{CL}=13.6$ |
| Meropenem | Isla  2008  n=20 | 18307371 | 2 | 6.63 L/h | 15.7 L | 15 L/h | 19.8 L | Exponential | Add, Prop | NONMEM | FOCE+I | $CL=\theta_{CL}\times\theta_{CLCR\_CL}\times{CL}_{CR}$  $\theta_{CL}=6.63$ $\theta_{CLCR\_CL}=0.064\left( septic \right)or 0.72(polytraumatized)$  $V1=\theta_{V1}$  $\theta_{V1}=15.7\left( septic \right)or 69.5 (polytraumatized)$  $C_{u=}C_{p}\times Sc$  $C_{u=}concentration in dialysate-ultrafiltrate$  $Sc=0.72 \left( 0.18 \right)sieving coefficient$ |
| Meropenem | Muro  2011  n=68 | 21366653 | 1 | 11.1 L/h | 33.6 L | NA | NA | Exponential | Add | NONMEM | FO | $CL=\theta_{CL}\times\left( \frac{mSCR}{0.7} \right)^{\theta_{mSCE\_CL}}$  $\theta_{CL}=11.1 \theta_{mSCR\_CL}=-1$ (if SCR < 0.4 mg/dL, mSCR=0.4) |
| Meropenem | Li  2006  n=79 | 16988206 | 2 | 14.6 L/h | 10.8 L | 18.6 L/h | 12.6 L/h | Exponential | Add, Prop | NONMEM | FO | $CL=\theta_{CL}\times\left( \frac{CLCr}{83} \right)^{\theta_{CLCr\_CL}}\times\left( \frac{AGE}{35} \right)^{\theta_{AGE\_CL}}$  $\theta_{CL}=14.6 \theta_{CLCr\_CL}=0.62 \theta_{AGE\_CL}=-0.34$ Cockcroft-Gault  $V1=\theta_{V1}\times\left( \frac{BW}{70} \right)^{\theta_{BW\_V1}}$  $\theta_{V1}=10.8 \theta_{BW\_V1}=0.99$ |
| Ciprofloxacin | Khachman  2011  n=102 | 21653603 | 2 | 18 L/h | 38 L | 60 L/h | 73 L | Exponential | Prop | NONMEM | FOCE-I | $CL=\theta1\times{{(CL}_{Cr Cockcroft}/91.7)}^{\theta2}$ |
| Ciprofloxacin | Conil  2008  n=70 | 18768301 | 2 | N | 62 L | NA | NA | NA | Prop | Kinetica | NA | $kel={CL}_{Cr}\left[ Cockcroft \right]\times0.0010203+0.119655$(h^-1^)  $V1=62$ (L)  $k_{12}=0.499$ (h^-1^)  $k_{21}=0.12$ (h^-1^) |
| Ciprofloxacin | Roberts  2015  n=24 | 25881576 | 1 | 58  ml/min | 37.7 L | NA | NA | Exponential | Add | NONMEM | FOCEI | NA |
| Ceftriaxone | Garot  2011  n=54 | 21545483 | 2 | 0.56 L/h | 10.3 L | 5.28 L/h | 7.35 L/h | Exponential | Prop | NONMEM | FOCE | $CL=\theta_{CL}+\theta_{CLCr}\times\frac{CLcr}{4.26}$Cockcroft-Gault (as instead)  $\theta_{CL}=0.56 \theta_{CLCr}=0.32$ |
| Vancomycin | Mangin  2014  n=30 | 25117184 | 2 | 1.91 L/h | 21.9 L | 5.71 L/h | 68 L | Exponential | Add | Monolix | SAEM | $CL=\theta_{CL}\times\theta_{FEMALE}\times\left( \frac{BW}{70} \right)^{0.75}\times\left( \frac{SAPSII}{70} \right)^{\theta SAPSII\_CL}\times\left( \frac{SCr}{70} \right)^{\theta SCr\_CL}$  $\theta_{CL}=1.91 \theta_{FEMALE}=0.66 \theta_{SAPSII\_CL}=-0.50 \theta_{SCr\_CL}=-0.90$  $V1=\theta_{V1}\times\left( \frac{BW}{70} \right)^{1}$  $\theta_{V1}=21.9$  $Q1=\theta_{Q1}\times\theta_{DIABETES\_Q1}\times\left( \frac{BW}{70} \right)^{0.75}$  $\theta_{Q1}=21.9 \theta_{DIABETES\_Q1}=0.30 \left( Diabetes \right) or 1 (non Diabetes)$  $V2=\theta_{V2}\times\left( \frac{BW}{70} \right)^{1}$  $\theta_{V1}=68$ |
| Vancomycin | Udy  2013  n=81 | 23473944 | 1 | 2.9 L/h | 0.8L/kg | NA | NA | Exponential | Add, Prop | NONMEM | FOCE+I | NA |
| Vancomycin | Revilla  2010  n=191 | 20653673 | 1 | 0.67 mL/min/kg | 0.82 L/kg | NA | NA | Exponential | Add | NONMEM | FOCE+I | $CL=\theta_{CL}\times{CL}_{Cr}\times{AGE}^{\theta AGE}$  $\theta_{CL}=0.67 \theta_{AGE\_CL}=-0.24$  $V1=\theta_{V1}\times{{Cr}_{se}}^{A}$  $\theta_{V1}=0.82 A=0 ({Cr}_{se}\leq1 md/dL)$ or $A=1 ({Cr}_{se}>1 md/dL)$ |
| Vancomycin | Llopis-Salvia  2006  n=50 | 16958822 | 2 | 0.034 L/h | 0.414 L/kg | 7.48 L/h | 1.32 L/kg | Exponential | Add, Prop | NONMEM | FOCE | $CL=\theta_{CL}\times{CL}_{Cr}+\theta_{BW}\times BW$  $\theta_{CL}=0.034 \theta_{BW}=0.015$  $V1=\theta_{V1}\times BW$  $\theta_{V1}=0.414$  $Q1=\theta_{Q1}$  $\theta_{Q1}=7.48$  $V2=\theta_{V2}\times BW$  $\theta_{V2}=1.32$ |
| Vancomycin | Roberts  2011  n=206 | 21402850 | 1 | 4.58 L/h (0.389) | 1.53 L/kg (0.374) | NA | NA | Exponential | Add, Prop | NONMEM | FOCE+I | $CL=\theta_{CL}\times\frac{{CL}_{Cr}}{100}$  $\theta_{CL}=4.58$  $V1=\theta_{V1}\times BW$  $\theta_{V1}=1.53$ |
| Vancomycin | Medellin-Garibay  2017  N=54 | 28893792 | 1 | 2.88 L/h | 1.03 L/kg | NA | NA | Exponential | Add | NONMEM | FOCE+I | $CL=\theta_{CL}\times\left( \frac{{CL}_{Cr}}{100} \right)^{\theta CLCr\_CL}\times\theta_{MV}$  $\theta_{CL}=2.86 \theta_{CLCr}=0.75 \theta_{MV}=0.8$ with mechanical ventilation, 1 without ventilation  ${CL}_{Cr}$=Cockcroft–Gault formula ml/min  $V1=\theta_{V1}\times BW$  $\theta_{V1}=1.03$ |

LEGEND

Add = Additive residual error

CL = Clearance of central compartment

CMT = Number of compartments

FO = First order estimation

FOCE = First order conditional estimation

IIV = Inter individual variability

n = Number of patient used for model development

NA = Not applicable

NCA = Non compartmental analysis

NPAG = Non Parametric Adaptive Grid

Prop = Proportional residual error

Q1 = Inter-compartmental clearance between central and peripheral compartments

V1 = Volume of distribution of central compartment

V2 = Volume of distribution of peripheral compartment

**Model validation method**

Retrospective and prospective pharmacokinetic data, including dosing information, demographic characteristics and biochemical measurements were extracted from the Amsterdam UMC, location VUmc (VUmc) and OLVG electronic health record (EHR) system.

The population parameters were fixed at the values reported in the publications. Predicted concentrations (PRED) were calculated at sampling times identical to those of our samples using NONMEM software. The metrics for predictive performance including mean error (ME), mean absolute error (MAE) and root mean squared error (RMSE) were calculated using observed and predicted values.

$$ME=\frac{1}{n}\sum_{i=1}^{n} \left( C_{{pred}_{i}}-C_{{obs}_{i}} \right) MAE=\frac{1}{n}\sum_{i=1}^{n} \left| C_{{pred}_{i}}-C_{{obs}_{i}} \right|$$

$$RMSE=\sqrt{\frac{1}{n}\sum_{i=1}^{n} \left( C_{{pred}_{i}}-C_{{obs}_{i}} \right)^{2}} PE=\frac{C_{pred}-C_{obs}}{C_{obs}}\times100\%$$

Goodness of fit plots is presented and in case of multiple candidate models prediction error (PE) was calculated and presented in bar graph.

**Model validation results**

Ceftriaxone

Only one model for Ceftriaxone dosing for ICU patients was found in the literature review. The model developed by Garot et al. is a two-compartment model detailed as following:

$$CL=\theta_{CL}+\theta_{CLCR}\cdot\frac{CLCR}{4.26}$$

Here, $\theta_{CL}$ is the typical value of clearance; $\theta_{CLCR}$ is the influencing constant of creatinine clearance on total clearance; $CLCR$ is creatinine clearance. Patient demographics from the Garot et al. study and for the evaluated patients for model validation are shown in Table 2.

Supplementary Table 2. Demographic characteristics of the study populations

|  | Garot | VUmc |
| --- | --- | --- |
| Number of patients | 54 | 24 |
| Patient type | ICU patients | ICU patients |
| Age (mean) | 68 | 65 |
| Male/Female ratio | 2.6 | 1.2 |
| Race | Caucasian | Mostly caucasian |
| Creatinine clearance | 5.5-214 ml/min | 12.9-168 ml/min |
| Dosing Scheme | 1g or 2g, QD | 2g, QD |
|  |  |  |

As is shown in supplementary Table 2, from a pharmacokinetic point of view, the VUmc patient population is similar to the Garot patient population. The Garot model incorporates creatinine clearance as a covariate for total clearance (CL). As the range of VUmc patients’ creatinine clearance is within that of the Garot patients’ creatinine clearance, the Garot model was deemed sufficiently suitable for our clinical setting. Additionly, since no other candidate models were identified through literature review, the Garot model was chosen for initial implementation.

Meropenem

9 pharmacokinetic (PK) models for meropenem were included for model validation. Retrospective pharmacokinetic data, including dosing information and plasma levels were extracted from both VUmc and OLVG EHR system. A total of 10 patients were included for model validation.

Supplementary Table 3. Metrics of PK model performance for meropenem

| Model | ME (mg/L) | MAE (mg/L) | RMSE (mg/L) |
| --- | --- | --- | --- |
| Muro | -14.06 | 15.24 | 91.13 |
| Frippiat | -15.82 | 24 | 102.49 |
| Jarura | -19.44 | 19.96 | 126.01 |
| Li | -22.8 | 22.82 | 147.74 |
| RamonLopez | -20.65 | 20.99 | 133.83 |
| Roberts2009 | -36.99 | 36.99 | 239.69 |
| Roberts2015 | -32.42 | 32.42 | 210.12 |
| Shekar | -27.51 | 27.98 | 178.31 |
| Tsai | -18.89 | 19.42 | 122.42 |


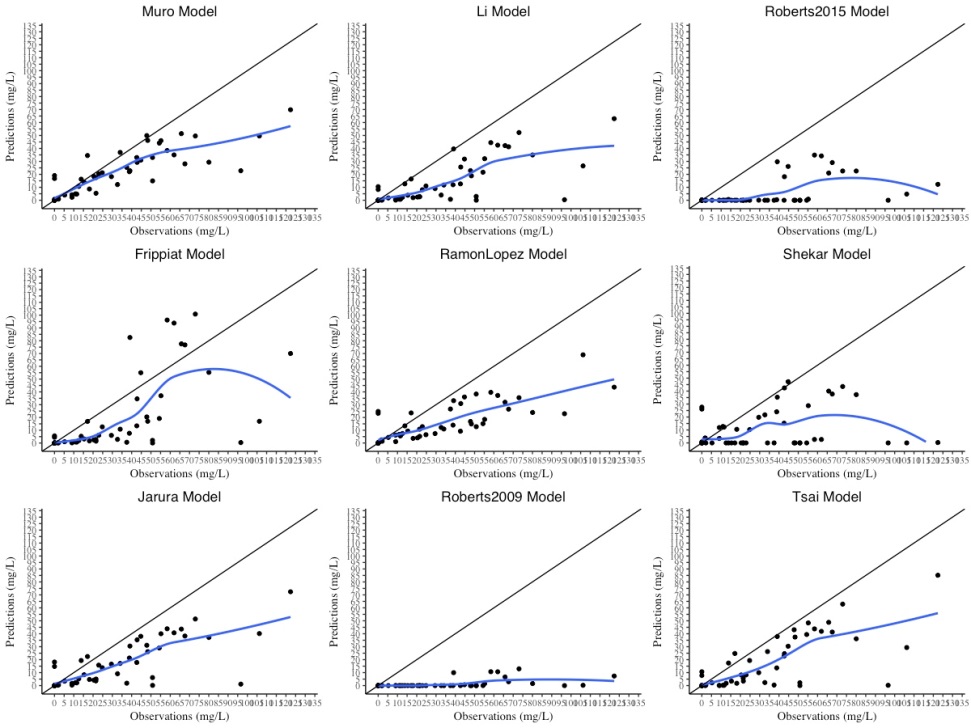


Supplementary Figure 1. Goodness of fit plot of the observed versus predicted concentration for candidate PK models for meropenem


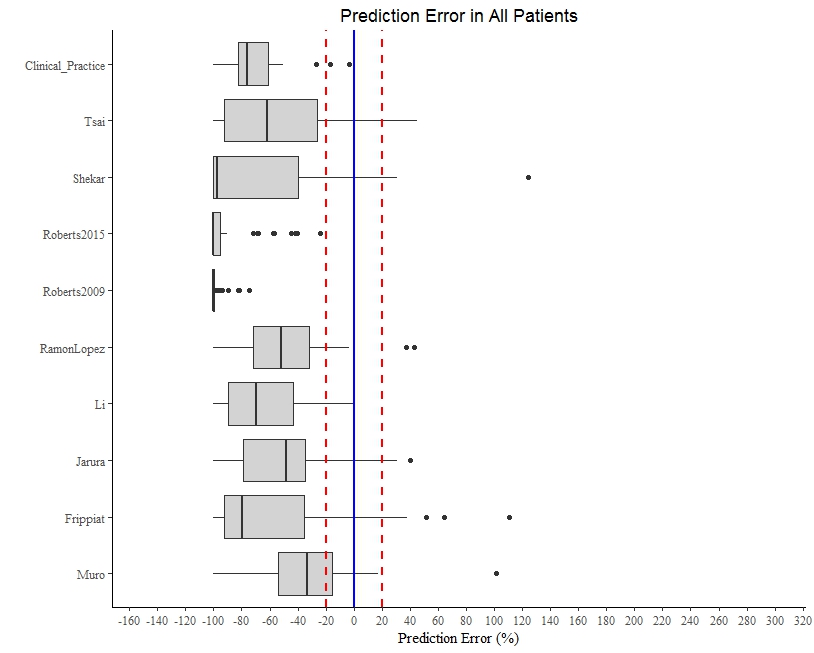


Supplementary Figure 2. Prediction error of candidate PK models for meropenem

Overall, nine candidate models for meropenem showed a measure of bias in our clinical data. However, a few of these models fit our data better than current clinical practice (supplementary Figure 2), meaning dosing based on current clinical protocol. Among candidate models, the Muro model performed best followed by the Jarura model and the RamonLopez model, see Supplementary Table 3. The Roberts2009 model and Roberts2015 model did not fit our data well and showed a very significant bias, see supplementary figure 1 and 2. In comparison to current clinical practise, see supplementary figure 2, some models performed similarly while some models, for example the Muro model, were significantly better at predicting concentrations. The Muro model predicted plasma concentration quite close to real observation, and with relatively small deviation. This model incorporates so-called ‘modified serum creatinine’ level, which substitutes all serum creatinine value lower than 0.4 mg/dL with 0.4 mg/dL. This could partially explain the slight underestimation of drug plasma concentration. The RamonLopez model fitted second best to our model, and it doesn’t incorporate any creatinine-related covariate, and therefore could be considered as an alternative model if no data is available. It is clear that remaining models did not perform well, according to the metrics calculation, goodness of fit and prediction error plots, see supplementary figure 1 and 2. As a result, the Muro model is selected for implementation in AutoKinetics.

Ciprofloxacine

3 PK models for ciprofloxacin were included for model validation. Retrospective pharmacokinetic data, including dosing information and plasma levels were extracted from both VUmc and OLVG EHR systems. A total of 12 patients were included for model validation.

Supplementary Table 4. Metrics of Model Prediction

| Model | ME (mg/L) | MAE (mg/L) | RMSE (mg/L) |
| --- | --- | --- | --- |
| Conil | 1.19 | 1.58 | 9.98 |
| Khachman | 0.52 | 1.22 | 4.37 |
| Roberts | 6.49 | 6.49 | 54.29 |


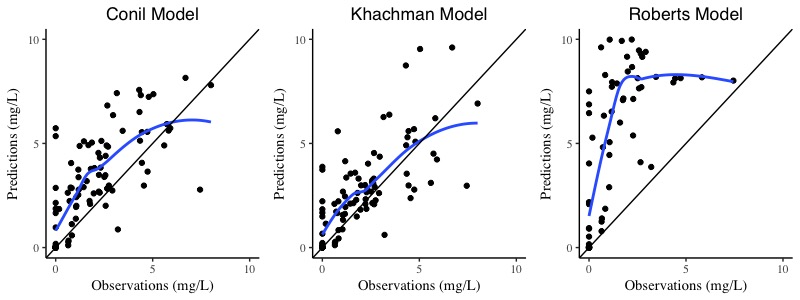


Supplementary Figure 3. Goodness of fit plot of the observed versus predicted concentration for candidate PK models for ciprofloxacin


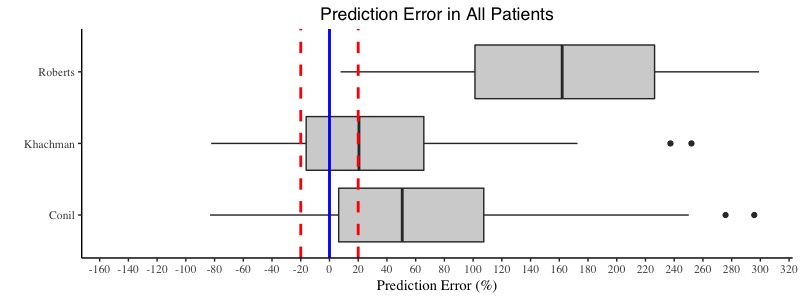


Supplementary Figure 4. Prediction error of candidate PK models for ciprofloxacin

See Supplementary table 4 for the model performance. Among the three candidate models, the Khachman model had the best predictive performance, followed by the Conil model. The Roberts model did not show an acceptable performance. The Conil model resulted in a similar MAE to that of Khachman model, but with a higher RMSE. Supplementary Figure 3 shows the goodness of fit (GOF) plot of the three candidate models. It is apparent that the Roberts model does not adequately fit the data. The Khachman model was the best fit to our data according to both goodness of fit and and prediction error graphs, see supplementary figure 3 and 4. Therefore, the Khachman model was selected for implementation.

**Cefotaxime model development**

A literature review was performed to identify candidate PK models for cefotaxime for the critically ill population. No PK models were found. We therefore developed a model on retrospectively collected pharmacokinetic data from the OLVG EHR system. 50 patients were included for model development.

Model development was carried out using nonlinear mixed effects modelling NONMEM (7.4.1). The first order conditional estimation method with interaction (FOCE+I) was used for modelling. One-, two- and three- compartment structural models with zero-order infusion and first-order elimination were evaluated as potential structural models. Inter-individual variability (IIV) and residual errors were analysed as well. IIV was assumed to follow a log-normal distribution and was described by the equation below:

$$P_{i}=P_{TV}\cdot e^{\eta i}$$

where $P_{i}$ is the parameter of ith individual; $P_{TV}$ is the typical value of the corresponding parameter, and $\eta$ represents the random variability of the parameter, which was assumed to be normally distributed with mean of 0 and variance of $\omega^{2}$. Full omega blocks and diagonal omega blocks were both evaluated. The residual error was described by both additive and proportional model, as following:

$$Y_{obs}=Y_{pred}\cdot\left( 1+\varepsilon_{prop} \right)+\varepsilon_{add}$$

where $Y_{obs}$represents the observation i.e. cefotaxime concentration; $Y_{pred}$ represents model-predicted cefotaxime concentration; $\varepsilon_{prop}$ and $\varepsilon_{add}$ represents the proportional residual error and additive residual error respectively. $\varepsilon_{prop}$ and $\varepsilon_{add}$ were assumed to follow a normal distribution with mean of 0, and variance of $\sigma_{prop}^{2}$, $\sigma_{add}^{2}$.

Base model was selected based on objective function and a number of goodness-of-fit plots. Covariates effects were analysed through forward selection and backward elimination procedure. Age, body weight, BMI, serum creatinine level, bilirubin, albumin, SAPSII, sepsis status, SOFA, CVVH treatment, fluid balance were evaluated. Correlations between continuous covariates and pharmacokinetic parameters were assessed using linear proportional models and power models. They were parameterised so that the covariate was centred on the mean value in the population. Diagnostic plots and decrease of objective function value were used to select covariates that improved the model prediction. A decrease in the objective function value of at least 10.83 (P<0.001) with one degree of freedom, relative to the base model was required for adding a single covariate to the model.

A one-compartment model best described the data. A full omega block was modelled because of a better fit when correlation between IIV was included. Albumin, SOFA score and serum creatinine level were identified as covariates that influenced clearance. No covariate was found that influenced volume of distribution. The full model is as follows:

$CL=\theta_{CL}\cdot\left( \frac{SCR}{100} \right)^{\theta_{SCR}}\cdot\left( 1+\theta_{ALB}\cdot\left( ALB-30 \right) \right)\cdot\left( 1+\theta_{SOFA}\cdot\left( SOFA-6 \right) \right)$

where $\theta_{CL}$ is the typical value of clearance, SCR is serum creatinine level (µmol/L), ALB is albumin level (g/L), SOFA is SOFA score.

Parameter estimates were summarised in Supplementary Table 5. Parameters were well estimated that all RSE were within acceptable interval.

Supplementary Table 5. Parameter estimates for cefotaxime model development.

| Parameter | Estimate | RSE |
| --- | --- | --- |
| $\boldsymbol{\theta}_{\boldsymbol{CL}}$ | 12.3 L/h | 7% |
| $\boldsymbol{\theta}_{\boldsymbol{SCR}}$ | -0.292 | 23.7% |
| $\boldsymbol{\theta}_{\boldsymbol{ALB}}$ | 0.0352 | 21.7% |
| $\boldsymbol{\theta}_{\boldsymbol{SOFA}}$ | -0.0412 | 27.9% |
| $\boldsymbol{\theta}_{\boldsymbol{V}}$ | 41.4 L | 7.5% |
| $\boldsymbol{\omega}_{\boldsymbol{CL}}^{\boldsymbol{2}}$ | 0.287 | 22% |
| $\boldsymbol{\omega}_{\boldsymbol{V}}^{\boldsymbol{2}}$ | 0.232 | 18% |
| $\boldsymbol{\omega}_{\boldsymbol{CL-V}}^{\boldsymbol{2}}$ | 0.23 | 19% |
| $\boldsymbol{\sigma}_{\boldsymbol{prop}}^{\boldsymbol{2}}$ | 0.108 |  |
| $\boldsymbol{\sigma}_{\boldsymbol{add}}^{\boldsymbol{2}}$ | 0.653 mg/L |  |

Goodness-of-fit plot showed that the model fitted our data nicely. Conditional weighted residual error (CWRES) randomly fell around 0 indicating that the model was not biased, see Supplementary figure 5.

Supplementary Figure 5. Goodness of fit plot of the observed versus predicted concentration for the developed PK model for cefotaxime
